# Multiscale Weighted Permutation Entropy Analysis of Schizophrenia Magnetoencephalograms

**DOI:** 10.3390/e24030314

**Published:** 2022-02-23

**Authors:** Dengxuan Bai, Wenpo Yao, Shuwang Wang, Jun Wang

**Affiliations:** 1 School of Telecommunications and Information Engineering, Nanjing University of Posts and Telecommunications, Nanjing 210003, China; baidengxuan_168@163.com; 2 Smart Health Big Data Analysis and Location Services Engineering Lab of Jiangsu Province, School of Geographic and Biologic Information, Nanjing University of Posts and Telecommunications, Nanjing 210023, China; 3 School of Electronic Information, Nanjing Vocational College of Information Technolog, Nanjing 210023, China; wangsw@njcit.cn

**Keywords:** weighted permutation entropy, multiscale, magnetoencephalogram, schizophrenia

## Abstract

Schizophrenia is a neuropsychiatric disease that affects the nonlinear dynamics of brain activity. The primary objective of this study was to explore the complexity of magnetoencephalograms (MEG) in patients with schizophrenia. We combined a multiscale method and weighted permutation entropy to characterize MEG signals from 19 schizophrenia patients and 16 healthy controls. When the scale was larger than 42, the MEG signals of schizophrenia patients were significantly more complex than those of healthy controls (p<0.004). The difference in complexity between patients with schizophrenia and the controls was strongest in the frontal and occipital areas (p<0.001), and there was almost no difference in the central area. In addition, the results showed that the dynamic range of MEG complexity is wider in healthy individuals than in people with schizophrenia. Overall, the multiscale weighted permutation entropy method reliably quantified the complexity of MEG from schizophrenia patients, contributing to the development of potential magnetoencephalographic biomarkers for schizophrenia.

## 1. Introduction

Approximately 0.32% of the world population (1 in 300 people) suffers from schizophrenia [1]. Schizophrenia is a very serious chronic disease that affects how a person thinks, feels, and behaves [2,3]. Its symptoms can be divided into three categories, namely positive symptoms, negative symptoms and cognitive deficit symptoms [4]. Positive symptoms mainly include hallucinations, delusions, thinking disorders, dyskinesias and other psychotic behaviors; negative symptoms, which are mainly related to the disruption of normal emotions and behaviors, include flat affect, lack of passion in life, lack of willpower and poverty of spontaneous speech; cognitive deficit symptoms include poor executive function, inability to concentrate, poor working memory and other problems [2,5]. To date, the diagnosis of schizophrenia is mainly based on clinical observations and patients’ descriptions of their experiences [4]. Individual cases of schizophrenia that exhibit the same symptoms may have different objective genetic backgrounds and brain network characteristics; the underlying etiology and pathogenesis of schizophrenia are still unclear [4,6]. Treatment of schizophrenia has remained mostly unchanged for decades and usually focuses on relieving symptoms [7,8].

In recent years, the development of brain imaging technologies has facilitated research on the structure and function of brain areas related to schizophrenia. Such technologies include magnetoencephalogram (MEG), electroencephalogram (EEG), functional magnetic resonance imaging (fMRI), structural magnetic resonance imaging (sMRI), positron emission tomography (PET), and computed tomography (CT). Among these imaging modalities, MEG has not only high temporal resolution but also high spatial resolution. An MEG system can detect extremely weak biomagnetic signals generated in the brain, providing completely noninvasive human brain imaging detection technology that does not expose the subject to radiation. Moreover, MEG is less affected by the conductive medium than EEG, and there is no need to manually install individual electrodes for testing [9,10]. Based on the characteristics of MEG signals in patients with schizophrenia, researchers have determined a variety of techniques for analyzing MEG signals in this population. These techniques include traditional time-frequency domain analysis [2,11,12], neurofeedback [13,14,15,16,17], cerebral hemisphere asymmetry [10,18], event-related potentials [16,19,20], and brain connectivity analysis [15,17,18,21,22]. The human brain is a very complex nonlinear system, but it is difficult to use the aforementioned methods to extract information on nonlinear dynamics from the brain signals of patients with schizophrenia. To address this problem, researchers have started to investigate the complexity of brain signals. The higher the complexity of a system, the more disorderly and irregular it is [23]. Investigating the complexity of brain signals can effectively provide information on their nonlinear characteristics and has thus attracted increasing interest [24]. When exploring the complexity of EEG signals in patients with schizophrenia, Ibanez-Molina et al. [25], Tan et al. [26], and Thilakvathi et al. [27] found that patients with schizophrenia had greater complexity than controls. However, there are relatively few studies exploring the nonlinear complexity of MEG in patients with schizophrenia. Fernández et al. [28] used the Lempel-Ziv (LZ) algorithm to explore the resting-state MEG complexity of schizophrenia patients and found that they had higher signal complexity than controls. When analyzing the MEG signals of schizophrenia patients. When analyzing the MEG signals of schizophrenia patients, Brookes et al. [29] suggested that the task-induced change in complexity was larger in the patients than that in controls.

In information theory, entropy is an effective index of the complexity of time series. Since the concept of permutation entropy (PE) was introduced by Bandt and Pompe [30], it has been used in various fields as a classic informational parameter for measuring nonlinear complexity [31,32], and it has also been modified for specific applications. PE characterizes the complexity of time series by calculating the permutation regularity of vectors in phase space, which offers the advantages of simplicity, ease of implementation, fast calculation speed and robust resistance to noise [33,34]. As an extension of PE, weighted permutation entropy (WPE) [35] can efficiently mine the information hidden in the amplitude of time series to reflect their evolution over time. Multiscale characteristics are a general feature exhibited by time series of complex systems [36]. The dynamic characteristics of complex systems can be most effectively explored by considering multiple scales [37]. Multiscale entropy (MSE) proposed by Cost et al. [38,39], can facilitate the extraction of structural information hidden in the scale factors of time series [39,40]. Yao et al. [41] used multiscale approximate entropy and MSE to analyze the complexity of MEG signals in patients with depression. Bai et al. [42] successfully identified schizophrenia using multiscale recurrence information in MEG signals. By analyzing the multiscale information of time series, we can better understand the complexity revealed by the deconstruction of complex systems. Therefore, multiscale weighted permutation entropy (MSWPE) has been successfully used in other fields to explore the dynamic information of time series [43,44,45]. Accordingly, we hypothesize that the complexity of MEG signals as measured by multiscale WPE (MSWPE) can be used as a marker to identify schizophrenia.

To analyze the complexity of MEG signals in patients with schizophrenia and search for potential biomarkers, we employed the MSWPE algorithm to investigate the complexity of patients’ brain activity. The MSWPE values of MEG signals from 19 schizophrenia patients and 16 healthy controls were calculated to elucidate the differences between these groups in terms of MEG signal complexity. This paper is organized as follows: Section 2 presents the experimental results, Section 3 includes a discussion of the findings, Section 4 introduces the method employed in the experiment, and Section 5 lays out the conclusions of this investigation.

## 2. Results

When calculating the MSWPE values of the MEG signal in each channel, we calculated the embedding dimension (*m*) according to the false nearest neighbors (FNN) [46] algorithm, and we obtained the embedding delay (τ) using the C-C [47] algorithm. After calculating the embedding dimensions and delays required by the sequence of each channel, we set the embedding dimension to 4 and the embedding delay to 2 accordingly. The sequence length of each channel was 144,000. We chose 1 as the smallest scale factor and 100 as the largest, the step size was set at 1. The MSWPE values of each participant’s MEG signals in each channel were calculated at a given scale, and the average value was used as the participant’s MSWPE value. Finally, the average values of the patients and controls at a specific scale factor were calculated to obtain the MSWPE values of each group. The Kolmogorov–Smirnov test was used to test the MSWPE values at a single scale factor for each individual. The MSWPE values at a single scale factor satisfied the assumptions of normal distributions and homogeneity of variance.

**Figure 1:**
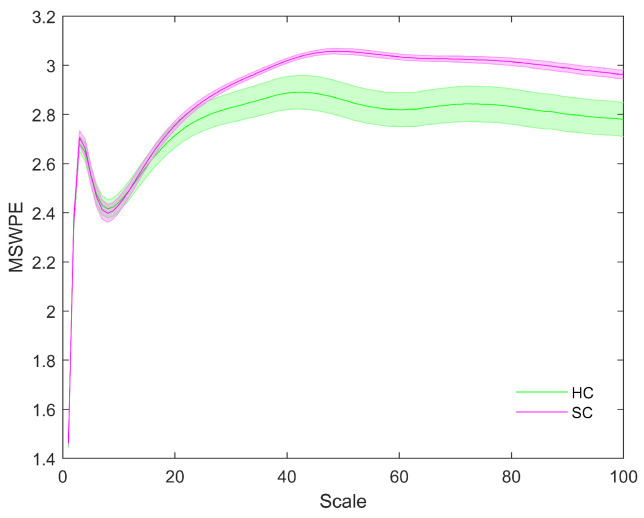
The MSWPE values (means ± standard errors) of the patients and the controls at different scales. HC in the legend represents the controls, and SC indicates the patients. The colored lines imply the mean MSWPE values in the two groups, and their shading signifies the standard error.

**Figure 2:**
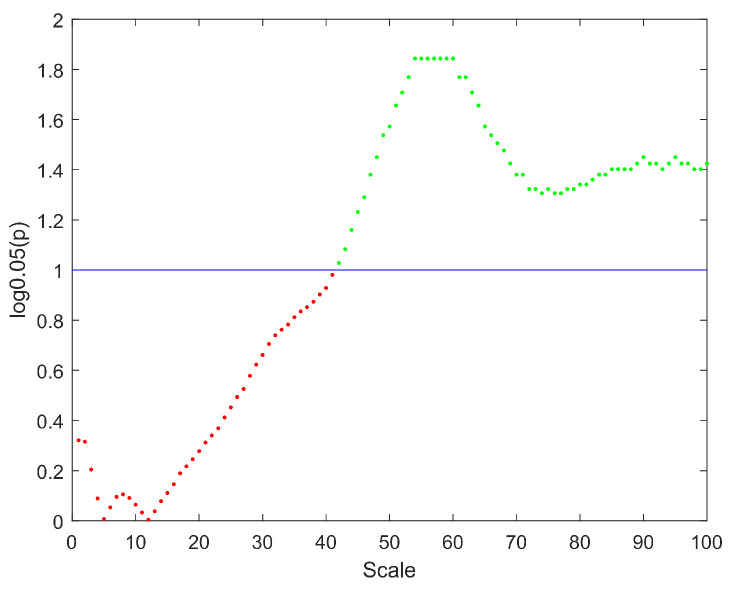
The distribution of *p* values at different scales. The y-axis represents the logarithm of *p* with base 0.05. The straight blue line implies the significance threshold of y=1. The scale factors without significant difference are denoted by red points, and the scale factors with significant difference are represented by green points.

The distributions of MSWPE values at each scale factor in the patients and controls are shown in Figure 1, in which the data are presented as the means and standard errors. An independent-samples *t*-test (p<0.05) was performed at each scale factor to test for a significant difference between the two groups, and the results are shown in Figure 2.

Figure 1 shows that the MSWPE values of the patients and controls followed consistent trends. When the scale factor increased, the MSWPE values first increased, then decreased, then increased to the maximum value, then slowly decreased and finally stabilized. This indicates that signals from the human brain have rich multiscale characteristics, with the dynamic evolution of these signals changing as the scale factor increases. When the scale factor was less than 20, the MSWPE values of the patients and controls were approximately equal. When the scale factor exceeded 20, the MSWPE values of the patients and controls started to differ, with the MSWPE values of the patients exceeding those of the controls. The distinction between the two groups became more obvious as the scale factor increased. Figure 1 clearly shows that the standard error of the controls was much larger than that of the patients. Figure 2 shows that there were statistically significant differences in MSWPE between the patients and controls when the scale factor exceeded 42 (represented by the green points in the Figure 2). Through analysis of the *t*-test results at each scale factor, We found the most significant difference between the two groups when the scale factor was 54–60.

**Figure 3:**
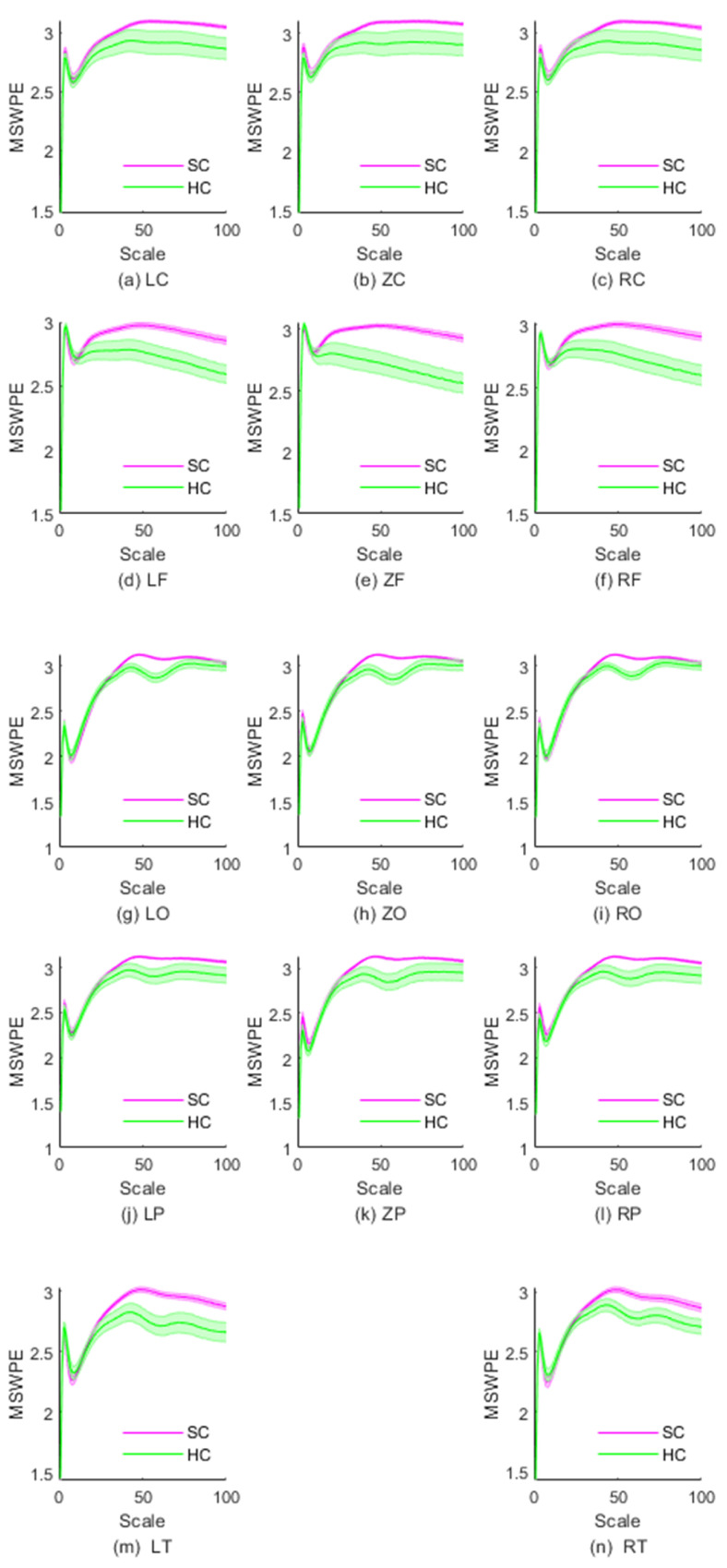
The MSWPE values (means ± standard errors) of different brain regions in SC and HC under different scale factors. HC denotes the healthy controls, and SC represents the patients with schizophrenia.

To further explore the differences in MEG complexity of schizophrenia patients, we studied the MSWPE values of different brain regions. The distribution of MSWPE values in each brain area is shown in Figure 3.

As is evident in Figure 3a–c, the MSWPE values of the LC, ZC, and RC regions exhibited a consistent trend. Figure 3 indicates that when the scale factor increased to a certain value, the controls and patients differed in MSWPE values, which may be useful for discriminating between the two groups. By analyzing the *p* values under each scale factor in the three regions, we found that the *p* values of these scales fluctuated above 0.05. However, the RC area differed in that when the scale factor was 99, the *p* value was less than 0.05 (p=0.048), although the *p* values of the surrounding scale factors were all greater than 0.05. We believe that this difference is due to error. Therefore, we concluded that there were no significant differences in MSWPE values between the patients and controls under any scale factor in the LC, ZC, or RC.

From first glance at Figure 3d–f, it appears that the variation trends in MSWPE values of the LF, ZF and RF regions are approximately parallel overall. However, a closer look reveals differences, in that the discriminative value of the RF area was between those of the ZF area and the LF area. The *t*-tests indicated that in the LF and RF areas, when the scale factor was 29, there was a significant difference in MSWPE values between the patients and controls and that the *p* values gradually decreased as the scale factor increased. In the ZF area, the *p* values began to decrease when the scale factor reached 24, and the decrease was relatively rapid. In all three regions, the larger the scale factor was, the smaller the *p* value. The ZF area had the smallest *p* values under the same scale factor.

As shown in Figure 3g–i, we found that the MSWPE values of the patients and controls ceased to be approximately equivalent at a scale factor of approximately 50 in the LO, RO, and ZO regions. The MSWPE values in the controls first showed a significant decline, then rebounded at a scale factor of 50. Additionally, in the controls, these areas had smaller standard errors than the other areas. According to the results of the *t*-tests, when the scale factor was 40, the *p* values of the LO and RO regions started to drop below 0.05, reaching 0.048 and 0.039, respectively. In the ZO area, when the scale factor was 39, the *p* value was less than 0.05. When the scale factor exceeded 72, the *p* values of all three regions started to exceed 0.05, reaching 0.043, 0.031 and 0.047 in LO, RO and ZO, respectively. The differences between the patients and controls in those three regions were most significant when the scale factor ranged from 50 to 62.

Figure 3j–l show that the variation trend of MSWPE values in the LP, ZP and RP regions at various scales was similar to the trend in the LO, ZO and RO regions. Nevertheless, in the LP, ZP, and RP regions, the controls had larger standard errors than those in the LO, ZO and RO regions, which indicates that the difference between the patients and controls diminished. By analyzing the t-test results, we found that when the scale factor changed from 46 to 64, there was a significant difference in the LP region between the patients and controls. The *p* values under the scale factors of 46 and 64 were 0.044 and 0.046, respectively. In the ZP region, all *p* values were less than 0.05 when the scale factor ranged from 41 (p=0.048) to 66 (p=0.046). In the RP region, when s∈[44,65], significant differences were observed between the patients and controls, and the *p* values under the scale factors of 44 and 65 were 0.039 and 0.045, respectively. The differences between the patients and controls in all three parietal regions were most significant when s∈[51,58].

According to Figure 3m,n, the changes in MSWPE values in the LT and RT areas were approximately consistent, but the standard errors in the LT area were larger, and the difference between the patients and controls was also more significant in the LT area than in the RT area. From the results of the *t*-test, we found that when s=41 (p=0.048), the *p* values in the LT area started to drop below 0.05; the *p* values in the RT area crossed below the threshold of 0.05 when s=44 (p=0.048). In these two regions, the difference between the patients and controls was most significant when the scale factor ranged from 54 to 60, with *p* values as low as 0.002.

In short, from Figure 3, it can be seen that the complexity of the patients and controls did not differ in the central area, but there were differences in other brain areas, especially in the frontal area and the occipital area. In Table 1, we present a simple summary of the results of t-tests in different brain regions.

It can be clearly seen from the Table 1 that the differences between the controls and patients were most significant in the frontal and occipital areas within the optimal ranges of scale factors, while there was no significant difference between the groups in the central area at any scale.

**Figure 4:**
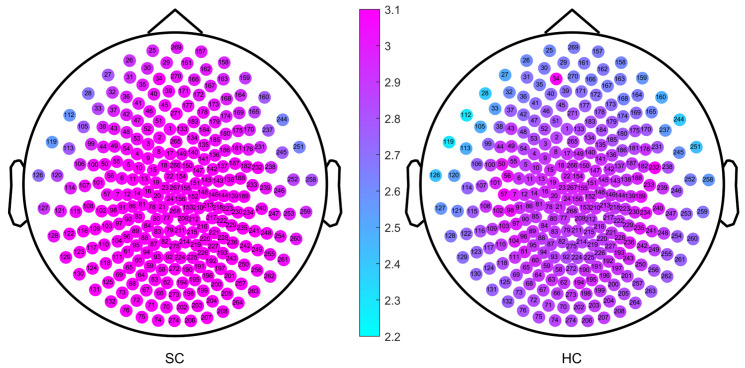
The MSWPE value distribution diagram of each channel in the patients and the controls under a scale factor of 57. HC denotes the healthy controls, and SC represents the patients with schizophrenia.

As part of the experimental design, we first calculated the single-channel MSWPE values of each participant under a certain scale factor and then calculated the mean across all channels to obtain the MSWPE values. Finally, we averaged the MSWPE values of all participants in a group to obtain the MSWPE values of the patients and controls. To determine whether the first averaging operation had any effect on the accuracy of the experimental results, we calculated the MSWPE values of each channel in the patients and controls under a single scale factor; we found that the results obtained under a single scale factor are consistent with the results shown in Figure 2 and Figure 3. Hence, we present only the experimental results under a scale factor of 57. These results are shown in Figure 4. The experimental results under other single scale factors will not be presented. Therefore, we believe that the first averaging operation of the previous experiment had a negligible effect on the accuracy of the experimental results.

**Figure 5:**
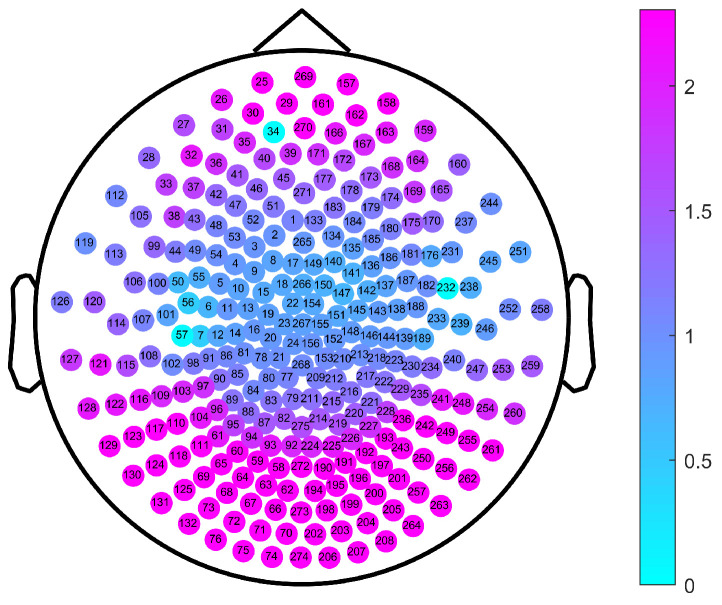
The *p* value distribution of channels for a comparison between the patients and the controls under a scale factor of 57. The color of the note represents the logarithm values of *p* with base 0.05.

The channels labeled 34 and 232 (corresponding to channels LF31 and RT12 of the Canadian CTF/VSM 275-channel full-head MEG system, respectively) were faulty; therefore, we set their MSWPE values to the maximum value of 5, as shown in Figure 4. It can be clearly seen from Figure 4 that when the scale factor was 57, the MSWPE values of the patients were considerably higher than those of the controls. In Figure 4, the patients and controls also showed significant differences in MSWPE values in the central area, which contrast with the results shown in Figure 3. The explanation of the conflicting results is that Figure 4 shows only the mean MSWPE values of a single channel for patients and controls, which do not reflect the size of standard errors. Therefore, we performed an independent-samples t-test on the MSWPE values of each channel for the two groups; the *p* value distribution for each channel is shown in Figure 5. Similarly, we set the *p* values of the two faulty channels to 1. As shown in Figure 5, all channels in the central area, some channels near the central area, and some channels in the temporal area had smaller logarithmic values. Therefore, there was no statistically significant difference in MSWPE values between the patients and the controls in these channels. However significant differences were presented on observed in other channels. At the same time, it can be clearly seen from Figure 5 that the difference in the MSWPE values between the patients and controls was the most significant in the frontal and occipital areas. These findings are consistent with the results shown in Figure 3.

## 3. Discussion

Our experimental results have led us to the following three main conclusions. First, schizophrenia patients and controls had similar MEG signal complexity under low scale factors, but under high scale factors, patients with schizophrenia exhibited higher complexity than controls. This suggests that brain activity of schizophrenia patients is more disordered and irregular than that of controls and is characterized by a high degree of whole-brain desynchronization. The difference in complexity between schizophrenia patients and controls was the most significant in the frontal and occipital areas, and there was almost no difference in the central area. By investigating the literature on different aspects of schizophrenia, we concluded that the above results may be related to disorders of the brain (such as abnormalities in functional connectivity [18,22], the synchrony of oscillations [48,49], or structure [50,51]) in patients with schizophrenia. As a result of this disordered brain activity, the communication between brain neurons is abnormal in schizophrenia patients, which increases the complexity of their MEG signals [25,26,27,28]. Furthermore, we also found that the standard errors of the controls were relatively large. The reason, we infer, is that healthy people have a wider dynamic range of brain activity than schizophrenia patients. These results are consistent with those found in other fields; for example, Ivanov et al. [52] demonstrated that healthy people have a wider dynamic range of heartbeat activity than unhealthy people. This finding provides good support for the plausibility of our inference.

A recent review of literature on the nonlinear complexity of brain signals in patients with schizophrenia identified several studies [25,26,27] whose conclusions are consistent with ours. Of course, there are also conflicting results regarding the complexity of brain signals in patients with schizophrenia; for example, Akar et al. [53] proved that schizophrenia patients had lower signal complexity than controls. In response to these conflicting results, we analyzed the two studies in search of an explanation. We found that the schizophrenia patients recruited in the work of Akar were older and had a longer disease duration and treatment period than our schizophrenia group. Therefore, we infer that the complexity of brain signals in patients with schizophrenia may be related to disease duration, age and pharmaceutical treatment. When studying the complexity of brain signals in schizophrenia, Raghavendra et al. [54] found that the complexity of signals increased in patients with positive symptoms and decreased in patients with negative symptoms. Lee et al. [55] revealed that the complexity of brain signals in schizophrenia was related to drug treatment. Fernández et al. [28] reported that the complexity of brain signals was related to the age of the individual. These findings provide good support for our inference.

Although MSWPE can be used to effectively identify schizophrenia patients by the characteristics of their MEG signals, our work still had some limitations. The first and greatest limitation is that the sample of MEG data was too small. Second, there is still no research on the difference in MEG signals in different frequency bands. Finally, we explored the MEG signals only in the resting state and did not investigate different stimulation regimens or different task states. Therefore, in future studies, we will include a larger sample size and investigate the complexity of MEG in the source space. Exploring MEG signals during different states and studying differences in a variety of frequency bands are also suitable directions for future research.

## 4. Materials and Methods

### 4.1. Participants

Nineteen schizophrenia (SC) patients (age: 25±8.322, median=23, mode=23) with positive symptoms and a healthy control (HC) group of 16 subjects (age: 25.6±5.152, median=24, mode=23) participated in our investigation. The SC patients were recruited from the outpatient clinic of the Affiliated Brain Hospital of Nanjing Medical University by psychiatrists. All members of the patient group were diagnosed with schizophrenia by experienced doctors in the Psychiatric Department of the Affiliated Brain Hospital of Nanjing Medical University. The Positive and Negative Syndrome Scale (PANSS) [56] was used to assess the clinical status of patients with schizophrenia. The PANSS scores of the SC patients were 99.29±6.302 (median=98, mode=93). The HC group was recruited by psychiatrists from the Affiliated Brain Hospital of Nanjing Medical University through an advertisement on the home page of the hospital website. Participants were excluded from this study if they (1) suffered from other neurological diseases, (2) had suffered severe brain trauma causing loss of consciousness for more than five minutes, (3) had abnormal intellectual development, or (4) had a history of drug or alcohol abuse in the two weeks before MEG was performed. (5) Female participants were excluded if pregnant. All individuals signed an informed consent form after the detailed procedure of this study was explained.

### 4.2. MEG Recording

The acquisition and recording of MEG signals was carried out by experienced senior engineers from the MEG Room of the Affiliated Brain Hospital of Nanjing Medical University. Data acquisition was conducted using the Canadian CTF/VSM 275-channel full-head MEG system. Before the MEG data were collected and recorded, the participants were required to remove all wearable devices and accessories that might affect the electromagnetic signals. During the collection and recording of MEG signals, the participants were asked to enter an electromagnetically shielded room, lie flat on the test bed with their eyes open, put their head into the helmet-shaped sensor array, and settle into a resting state. After the participants were confirmed to be in a resting state, MEG signals were scanned and recorded continuously for 2 min. Then, the recorded MEG signals were digitized with a sampling frequency of 1200 Hz. Two sets of resting-state MEG signal data were recorded from each participant. As the MEG signals were recorded, each participant’s electrocardiogram (ECG) and electrooculogram (EOG) signals were recorded simultaneously to facilitate subsequent inspection of the MEG data. During the entire MEG signal recording period, the participants were instructed to not make any movements that would affect the accuracy of the data, including blinks and muscle movements. During the scanning process, the data acquisition engineer supervised the participants and provided instructions through the cameras and walkie-talkies installed in the electromagnetically shielded room. If the participant was found to have made a movement that might affect the accuracy of the results, the recorded signal was discarded, and a new recording was made.

### 4.3. Preprocessing of MEG Dataset

First, all MEG data were preprocessed offline in MATLAB2013 using the FieldTrip toolbox. We used the 0.1–200 Hz digital bandpass filter to filter the original data. The power noise generated by 50 Hz and its harmonics were processed by notch treatment. Then, the MEG data were manually checked (removing artifacts such as eye movement, heartbeat, etc.) by a neurophysiologist using ICA via EEGLAB toolbox; datasets with excessive interference were removed. Data from 2 SC patients and 1 HC individual were discarded due to excessive interference.

### 4.4. Multiscale Weighted Permutation Entropy

To obtain the MSWPE, two processes, i.e., the coarse-grained multiscale process and the weighted permutation entropy, were required.

#### 4.4.1. Coarse-Graining Process

Given a one-dimensional time series {x(i):i=1,2,⋯,N}, the multiscale process is to construct a coarse-grained time series. When the scale factor is 1, the coarse-grained series coincides with the original time series. When the scale factor is *s*, the constructed coarse-grained time series is {ys(j):j=1,2,⋯,NNss}, where $y^{s}\left(j\right)$ is calculated by the following Formula (1)

(1) ys(j)=1s∑i=(j−1)s+1jsx(i)(1≤j≤NNss)

with *s* representing the scale factor.

#### 4.4.2. Weighted Permutation Entropy

The phase space of the coarse-grained time series [38,39] can be reconstructed as follows Formula (2).

(2) Ys(n)={ys(n),ys(n+τ),⋯,ys(n+(m−1)τ)}(n∈[1,NNss−m+1])

where *m* is the embedding dimension, and τ is the embedding delay time. Then, the weight corresponding to each vector in the phase space can be calculated using the following Formula (3).

(3) $$\begin{array}{c} w\left(n\right)=\frac{1}{m}\sum_{j=1}^{m}{\left\{y^{s}\left[n+\left(j-1\right)\tau \right]-\bar{Y^{s}\left(n\right)}\right\}}^{2} \end{array}$$

where $\bar{Y^{s}\left(n\right)}$ is the arithmetic average of $Y^{s}\left(n\right)$, calculated by the following Formula (4).

(4) $$\begin{array}{c} \bar{Y^{s}\left(n\right)}=\frac{1}{m}\sum_{j=1}^{m}y^{s}\left[n+\left(j-1\right)\tau \right] \end{array}$$

Then, the *m* components of $Y^{s}\left(n\right)$ are arranged in ascending order as Formula (5).

(5) $$\begin{array}{c} y^{s}\left[n+\left(k_{1}-1\right)\tau \right]\leq y^{s}\left[n+\left(k_{2}-1\right)\tau \right]\leq \cdots \leq y^{s}\left[n+\left(k_{m}-1\right)\tau \right] \end{array}$$

If several components have equal values, they should be arranged in ascending order according to their *k* values. The indexes of equal values are modified to be the same as those in their corresponding groups, e.g., by the smallest or largest index [57]. In permutation entropy, ordinal patterns are labels of different vectors; therefore, the original permutation and amplitude permutation make no difference [57]. In this manner, every vector $Y^{s}\left(n\right)$ in the phase space can be hit to obtain a set of permutations $\pi_{n}=\left\{k_{1},k_{2},\cdots ,k_{m}\right\}$. The probability of occurrence of each permutation is calculated by Formula (6).

(6) ps(w,M)=∑n=1NNss−(m−1)τ1u:type(u)=πn(Ys(n))·w(n)∑n=1NNss−(m−1)τ1u:type(u)∈Π(Ys(n))·w(n)

According to the definition of Shannon entropy, the weighted permutation entropy [35] of time series at scale *s* is as Formula (7).

(7) $$\begin{array}{c} H^{s}\left(p,w\right)=-\sum_{M=1}^{m!}{p^{s}}_{\left(w,M\right)}\ln{p^{s}}_{\left(w,M\right)} \end{array}$$

When ps(w,M)=11m!m!, $H^{s}\left(p,w\right)$ reaches its maximum value ln(1ln(1m!m!). Usually, ln(1ln(1m!m!) is used to normalize the WPE, the normalized WPE can be calculated as follows Equation (8).

(8) hs(p,w)=Hs(p,w)ln(1ln(1m!m!)

### 4.5. Division of Brain Regions

**Figure 6:**
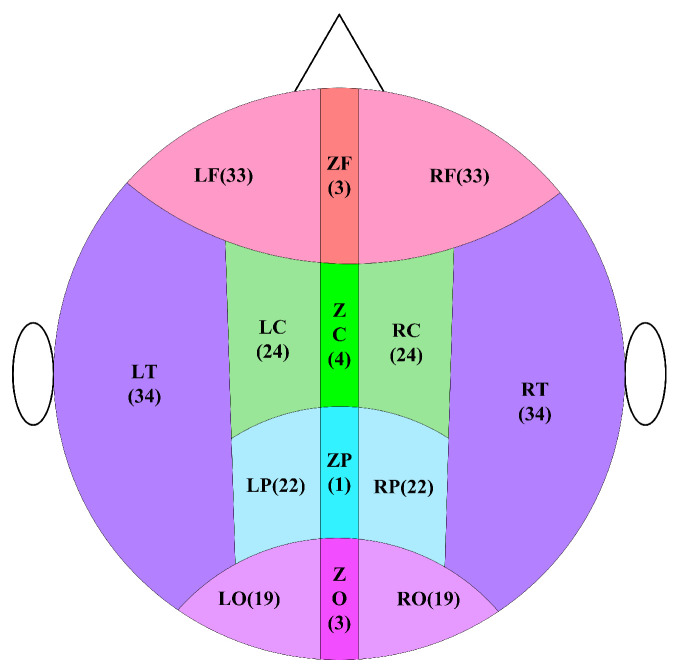
Schematic diagram of brain division. In the figure, the letter C represents the central area, the letter F means the frontal area, the letter O implies the occipital area, the letter P indicates the parietal area, and the letter T denotes the temporal area. In the subdivision of areas, the letter L signifies the left, the letter Z represents the middle, and the letter R defines the right. The number indicates the number of channels.

**Figure 7:**
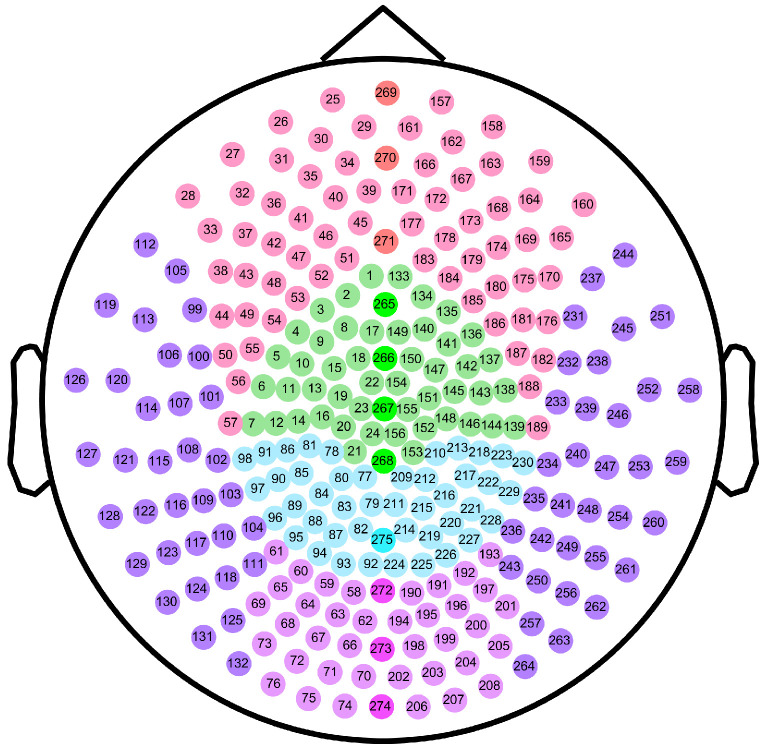
Schematic diagram of channel distribution in each brain area. The dots in the figure represent the channels of the Canadian CTF/VSM 275-channel full-head MEG system. The color of each dot indicates the brain area where the channel is located. The numbers represent our labels for the individual channels.

To further explore the differences in MSWPE values between the patients and the controls in different brain regions, the brain was divided into five regions (namely the central area (C), frontal area (F), parietal area (P), occipital area (O), and temporal area (T)) according to the distribution of the channels in the full-head MEG system. Every area other than T was further subdivided into three parts: left, middle, and right, and the T was divided into left and right parts. A diagram of the division of brain areas is shown in Figure 6, and the channel distribution of each brain area is shown in Figure 7.

### 4.6. Statistical Analysis

All statistical analyses were performed using the Statistical Product and Service Solutions (SPSS) software. The Kolmogorov–Smirnov test was used to test whether the data satisfied the assumptions of normal distribution and homogeneity of variances. Two-tailed independent-sample *t*-tests (p<0.05) were used to analyze the differences in complexity between the patients and controls.

## 5. Conclusions

In conclusion, we employed MSWPE to analyze the complexity of MEG signals in patients with schizophrenia. Schizophrenia MEGs have higher MSWPE than the healthy subjects, particularly when the scale factor is between 54 and 60. Moreover, the frontal and occipital MEG significantly differentiate the schizophrenia and the controls in statistics. MSWPE can be used as a marker of schizophrenia to facilitate the clinical diagnosis of the disease.

## Figures and Tables

**Table 1:** Summary of *t*-test results in different brain regions.

Brain Region	Scale Intervals with Significant Differences	Minimum Value of *p*
LC	No significant difference	p>0.05
ZC	No significant difference	p>0.05
RC	No significant difference	p>0.05
LF	[29, 100]	0.001
ZF	[24, 100]	p<0.001
RF	[29, 100]	0.001
LO	[40, 72]	p<0.001
ZO	[39, 72]	p<0.001
RO	[40, 72]	p<0.001
LP	[46, 64]	0.016
ZP	[41, 66]	0.006
RP	[44, 65]	0.009
LT	[41, 100]	0.002
RT	[44, 100]	0.002

## Data Availability

Restrictions apply to the availability of these data. Data was obtained from the Affiliated Brain Hospital of Nanjing Medical University and are available from Jun Wang with the permission of the Affiliated Brain Hospital of Nanjing Medical University.

## Abbreviations

The following abbreviations are used in this manuscript:

MEG	Magnetoencephalogram
EEG	Electroencephalogram
fMRI	Functional magnetic resonance imaging
sMRI	Structural magnetic resonance imaging
PET	Positron emission tomography
CT	Computed tomography
LZ	Lempel-Ziv
PE	Permutation entropy
WPE	Weighted permutation entropy
MSE	Multiscale entropy
MSWPE	Multiscale weighted permutation entropy
FNN	False nearest neighbors
SC	Schizophrenia patients
HC	Healthy control
ECG	Electrocardiogram
EOG	Electrooculogram
